# Recommendations for a standardised educational program in robot assisted gynaecological surgery: Consensus from the Society of European Robotic Gynaecological Surgery (SERGS)

**Published:** 2019-03

**Authors:** P Rusch, T Ind, R Kimmig, A Maggioni, J Ponce, V Zanagnolo, PJ Coronado, J Verguts, E Lambaudie, H Falconer, JW Collins, RHM Verheijen

**Affiliations:** Department of Obstetrics and Gynaecology, University Hospital Duisburg-Essen; Hufelandstr. 55, 45147 Essen, Germany. Email: peter.rusch@uk-essen.de. Email: rainer.kimmig@uk-essen.de;; Department of Gynaecological Oncology, The Royal Marsden, London, UK;; St George’s University Hospitals NHS Foundation Trust, Blackshaw Road, Tooting, London, Email: thomasind@rmh.nhs.uk;; Division of Gynaecology, European Institute of Oncology, Via Ripamonti, 435, 20141 Milano, Italy. Email: angelo.maggioni@ieo.it. Email: vanna.zanagnolo@ieo.it;; Department of Gynaecological Oncology, Hospital Universitari de Bellvitge, c/ Feixa Llarga, sn, 08907 L’ Hospitalet de Llobregat. Barcelona, Spain. Email: jponce@bellvitgehospital.cat;; Department of Gynaecological Oncology, Hospital Clínico San Carlos, Universidad Complutense de Madrid, Avda. de Séneca, 2, Ciudad Universitaria, 28040 Madrid, Spain. Email: pcoronadom@gmail.com;; Department of Obstetrics and Gynaecology, University Hospitals Leuven, 3000 Leuven, Belgium;; Department of Obstetrics and Gynaecology, Jessa Hospital, 3500 Hasselt, Belgium, Email: jasper.verguts@jessazh.be;; Department of Gynaecologic Oncology, Centre de Lutte Contre le Cancer Oscar Lambret, 3 Rue Frédéric Combemale, 59000 Lille, France;; Aix Marseille Université, Site Timone, Timone 27, boulevard Jean Moulin, 13385 Marseille cedex 5, France. Email: lambaudiee@ipc.unicancer.fr;; Department of Women’s and Children’s Health, Division of Obstetrics and Gynecology, Karolinska Institutet/University Hospital, 171 76 Stockholm, Sweden. Email: henrik.falconer@karolinska.se;; Department of Urology, Karolinska University Hospital, Karolinska Universitetssjukhuset, Solna, D1:01 171 76 Stockholm, Sweden. Email: justin.collins@ki.se;; Department of Gynaecological Oncology, UMCU Cancer Center, University Medical Center, Utrecht, Netherlands. Email: rene.h.m.verheijen@gmail.com

**Keywords:** Delphi, training, robot assisted surgery, consensus

## Abstract

**Background:**

The Society of European Robotic Gynaecological Surgery (SERGS) aims at developing a European consensus on core components of a curriculum for training and assessment in robot assisted gynaecological surgery.

**Methods:**

A Delphi process was initiated among a panel of 12 experts in robot assisted surgery invited through the SERGS. An online questionnaire survey was based on a literature search for standards in education in gynaecological robot assisted surgery. The survey was performed in three consecutive rounds to reach optimal consensus. The results of this survey were discussed by the panel and led to consensus recommendations on 39 issues, adhering to general principles of medical education.

**Results:**

On review there appeared to be no accredited training programs in Europe, and few in the USA. Recommendations for requirements of training centres, educational tools and assessment of proficiency varied widely. Stepwise and structured training together with validated assessment based on competencies rather than on volume emerged as prerequisites for adequate and safe learning. An appropriate educational environment and tools for training were defined. Although certification should be competence based, the panel recommended additional volume based criteria for both accreditation of training centres and certification of individual surgeons.

**Conclusions:**

Consensus was reached on minimum criteria for training in robot assisted gynaecological surgery. To transfer results into clinical practice, experts recommended a curriculum and guidelines that have now been endorsed by SERGS to be used to establish training programmes for robot assisted surgery.

## Introduction

The introduction of robotics in gynaecological surgery has resulted in a need for new surgical skills and a requirement for a syllabus, training structure and assessment model. Professional societies should, in parallel to training programs in conventional endoscopy (Campo et al., 2016), also develop programs for safe and efficient training in specific areas such as robotic surgery.

Laparoscopic surgery was introduced in the late sixties although it was not until recently that regulatory authorities realised that the traditional master-apprentice principle was insufficient to provide safe and adequate skills and to monitor proficiency (Stassen et al., 2010). Consequently, there has been criticism on the way surgeons are trained (Li et al., 2016; Sridhar et al., 2017; Beane et al., 2019). Furthermore, deficient training and credentialing predisposes to litigation (Lee et al., 2011). Trainees perceive that training in laparoscopic surgery, and in particular robotics, is poor (Gan et al., 2017). This has led to a call for more structured and validated training and more virtual instruction (Schreuder et al., 2012; Beane, 2019).

Although curricula for training in conventional laparoscopy are developing (Tremblay et al., 2014), this is evolving more slowly for robot-assisted surgery (Fisher et al., 2015). Nevertheless, the Society of American Gastrointestinal and Endoscopic Surgeons (SAGES), together with the Minimal Invasive Robotic Association (MIRA) drafted a position paper in 2007 (Herron et al., 2008). This resulted in the first curriculum, the Fundamentals of Robotic Surgery (FRS) in the USA (Smith et al., 2014). The European Board and College of Obstetricians and Gynaecologists (EBCOG) issued ‘Robotic Surgery Standards’ as part of their ‘Gynaecology Standards’ (Mahmood et al., 2014). This latter document describes training in broad terms only, but defines the learning curve of surgeons that should be ‘specifically trained’ for robot-assisted procedures.

Urologists were the first in Europe to propose a curriculum. The syllabus developed by the European Association of Urologists (EAU) Robotic Urology Section (ERUS) is the only curriculum that encompasses the complete pathway from technical instruction to patient procedures (Herron and Marohn, 2008). The Society of European Robotic Gynaecological Surgery (SERGS) also aimed to develop guidelines for the safe introduction of robot-assisted surgery although consensus was lacking on many issues (Rusch et al., 2018). A Delphi process which is described in this paper was necessary to finalise a curriculum that is proposed to be used for robotic gynaecological surgery.

## Materials and methods

In January 2017 an expert advisory committee was formed to formulate a consensus on recommendations for education in robotic gynaecological surgery. Fifteen experienced surgeons and members of SERGS were invited and eleven accepted. A fellow trainee (PR) was invited also, along with a member of the EAU (JWC) who had experience in guiding such process.

**Figure 1:**
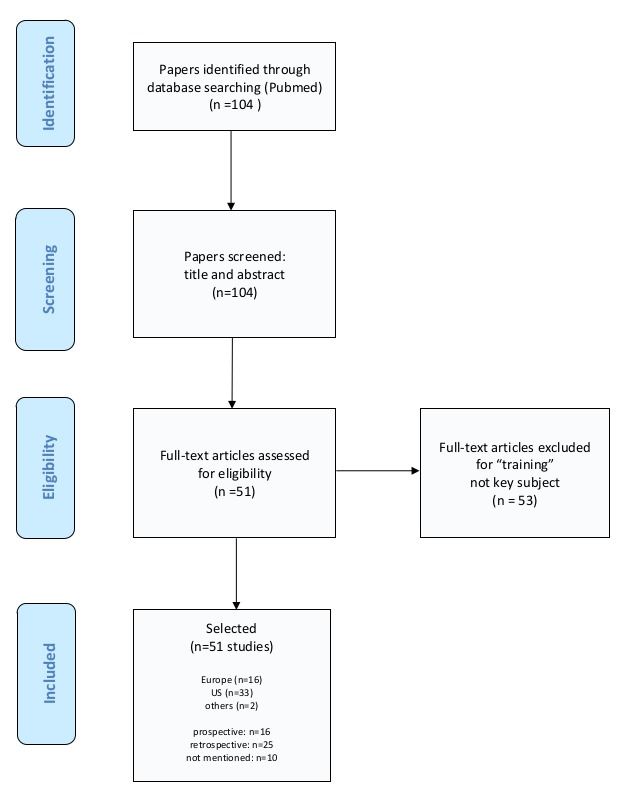
Selection process of papers for the literature review.

The strategy was divided into two parts. The first was a systematic literature review (Figure 1). A search was undertaken using Pubmed and Medline with the key terms “robotic”, “training”, “gynecology”, “surgery” AND “assisted”. Articles selected included single-centre series, meta-analyses, randomised controlled trials (RCTs) and systematic reviews between 2007 and 2017. The search yielded a total of 104 potential studies, of which 51 focused on training, testing or credentialing in robotic assisted gynaecological surgery. These papers were then screened for key questions divided in subgroups on four main subjects, (a) qualification/credentialing, (b) course/content of robotic training, (c) methodology/structure of robotic training and (d) testing/test instruments.

In the second phase, the literature review lead to formulating key questions for a Delphi survey (Thangaratinam, 2005). The aim was to achieve agreement on 39 aspects of a robotic curriculum emerging from this review and from the experts’ experience (Table I). An internet survey was generated and sent to panel members. The questionnaire was completed over three rounds. Google Forms® analytical software was used to record and measure consensus levels of the e-consensus at each round. Results were displayed as percentages so they could be reflected on before selecting a response in subsequent rounds. In the second and third rounds questions on which an 80% consensus was reached, were removed. Cronbach alpha was chosen as a measure of consistency. A cut-off value of 0.8 was chosen to determine consensus. After the three rounds a meeting was held to present results focusing on those questions that had not reached a 0.8-consensus. The final manuscript was reviewed and approved by the SERGS Council.

**Table I:** — Recommendations for a standardised educational programme in robot assisted gynaecological surgery: elements that reached 80–100% agreement on the Google form survey using the Delphi process.

No.	Question/Answer	Consensus
0. Curriculum – General Agreement		
1	Q: Do you agree that a standardised robotic training curriculum for gynaecology will be advantageous to robotic training?	A standardised robotic training curriculum for gynecology will be advantageous to robotic training (100%).
1. Qualification		
Trainer & Trainee		
2	Q: Experienced surgeons are exempt from completing the advanced procedural training assessment. But should learn about the basic training in new robotic systems, if they are using a new system?	Experienced surgeons are exempt from completing the advanced procedural training assessment; but they should learn about the basic training in new robotic systems, if they are using a new system (100%).
3	Q: Experienced surgeons should still study and be tested on the advanced robotic curriculum?	50%; failed
4	Q: What is the minimum number of cases that a trainee should be mentored/proctored by an experienced trainer before they are independent surgeons?	The minimum number of cases that a trainee should be mentored/proctored by an experienced trainer before they are independent surgeons is 10 cases (80%).
5	Q: Should trainers/proctors be assessed and certified?	Trainers/proctors should be assessed and certified (100%).
6	Q: Should surgeons continue to report their outcomes after ‘certification’ with a standardised reporting template?	60%; failed
7	Q: Should training centers be assessed and accredited via a recognised society?	Training centers should be assessed and accredited via a recognized society (100%).
8	Q: Should training centers be accredited related to case volume in the specialty via a recognised society?	Training centers should be accredited related to case volume in the specialty via a recognized society (80%).
9	Q: Should training centers be accredited related to the expertise of the trainers and the case volume in the robotic hospital affiliated with the training centre. If so how many cases/year are required?	Training centers should be accredited related to the expertise of the trainers and a case volume of >100 cases /year in the robotic hospital affiliated with the training centre (90%).
Reporting		
10	Q: Components of a standard reporting template should include which components?	Components of a standard reporting template should include patient specific details (80%), comorbidities (80%), BMI (80%), operation details (80%), length of stay (80%), pre-operative staging (80%), operation time (90%), pathological staging(80%, readmission rate (80%), Clavien-Dindo (80%).
2. Course/ Content of Curriculum		
11	Q: Should the curriculum be divided into stages?	The curriculum should be divided into stages (90%).
Basic Training		
12	Q: The basic robotic curriculum should include which parts/stages (can tick multiple answers as required)	Basic robotic curriculum should include baseline evaluation (90%), e-learning module (online access to information) (80%), simulation based training (100%), robotic theatre (bedside) observation (90); team simulation (90%).
13	Q: Baseline evaluation should include which parts/stages (can tick multiple answers as required).	Baseline evaluation should include VR simulation (90%) and written knowledge test (80%).
14	Q: E-learning should include which elements for basic training (can tick multiple answers as required)	E-learning should include designated elements for basic training: Information on patient selection (100%), Information on port placement (100%), How to dock the robot cart (100%), Trouble shooting (100%), Link to FRS (80%), Theatre team efficiencies (100%), Non-technical skills (90%), Standardized emergency management (90%)
15	Q: The required operating room observation should be:	The required operating room observation should be case number dependent (90%).
16	Q: Basic simulation training should include:	Basic simulation training should include VR simulation (100%), Dry lab training (100%, Wet-lab training (90%).
17	Q: Trainees should pass the basic training before commencing the advanced training?	Trainees should pass the basic training before commencing the advanced training (90%).
Advanced Training		
18	Q: Advanced robotic training should include?	Advanced robotic training should include e-learning on index procedures with video demonstration (100%), access to video library (100%), simulation training (90%), modular console training (90%), transition to full training (100%), final evaluation (90%).
19	Q: Advanced e-learning should include:	Advanced E-learning should include modular (stepwise) approach (100%), information on patient selection and preparation (100%), port placement (90%), non-technical skills training (90%), trouble shooting (100%), emergency scenario management information (100%), list of additional equipment that should be available in theatre (90%).
20	Q: Non-technical skills training should include.	70%; failed
21	Q: Team training should include.	Team Training should include emergency scenarios (80%), team decision making (80%), bedside assistance (90%), docking (90%) and patient turnaround (80%).
3. Structure of Curriculum		
Target Groups		
22	Q: Robotic curriculum training should take into account the experience of the different target groups to include (can tick multiple boxes)	Robotic curriculum training should take into account the experience of residents (100%), fellows (100%), robot naïve (100%), nurses (90%), lap surgeons (90%).
23	Q: Do you agree that there should be a common approach for basic robotic skills training with a similar pathway across subspecialty groups?	Agreement that there should be a common approach for basic robotic skills training with a similar pathway across subspecialty groups (90%).
Course/Sequence		
24	Q: Is a stepwise approach (modular training) to an index procedure advantageous to training?	A stepwise approach (modular training) to an index procedure is regarded advantageous (100%).
25	Q: Is an index procedure, which should be mastered within a given period of time, necessary?	An index procedure mastered within a given period of time is necessary (80%).
26	Q: If so, do you agree that for benign gynecology a suitable index procedure would be?	A suitable index procedure for benign gynecology would be benign hysterectomy (90%).
27	Q: If so, do you agree that for gynecology oncology a suitable index procedure would be?	A suitable index procedure for gynecological oncology would be pelvic lymphadenectomy (80%).
28	Q: Is a resident experienced trainer/proctor necessary when the trainee is proceeding to ‘transition to full procedure’ in the surgeons home institution?	A resident experienced trainer/proctor is necessary when the trainee is proceeding to “transition to full procedure” in the surgeons home institution (100%).
4. Test Instruments		
E-Learning		
	Q: Each section of the e-learning should have questions to evaluate knowledge.	Each section of the e-learning should have questions to evaluate knowledge (90%).
30	Q: Advanced e-learning modules should be evaluated with online tests?	Advanced E-learning modules should be evaluated with online tests (100%).
Evaluation, Analysis		
31	Q: Non-technical skills training should be evaluated with a scoring system?	Non-technical skills training should be evaluated with a scoring system (80%).
32	Q: Non-technical skills can be sufficiently assessed with NOTSS (Non-Technical Skills for Surgeons)?	Non-technical skills can be sufficiently assessed with NOTSS (80%).
33	Q: Would trainees benefit from validated scoring systems to provide more consistent feedback?	Trainees would benefit from validated scoring systems to provide more consistent feedback (90%).
34	Q: Should full procedure technique be evaluated with a submitted video to certified independent examiners?	Full procedure technique should be evaluated with a submitted video to certified independent examiners (80%).
35	Q: If answer to above yes, which case number should be sent for analysis and feedback?	70%, failed
36	Q: Evaluation of videos should be completed with a validated standardised scoring system?	Evaluation of videos should be completed with a validated standardized scoring system (80%).
37	Q: Scoring systems for video analysis should include (can tick multiple boxes)?	Scoring systems for video analysis should include a combination of subjective and objective scoring systems (e.g. GEARS, OSATS, a new objective scoring system) (100%).
38	Q: How many ‘experts’ should analyse the surgery videos?	2 experts should analyse the surgery videos (90%).
39	Q: Should video analysis and the logbook be the final evaluation step for ‘certification’?	Video analysis and the logbook should be the final evaluation step for certification (90%).

## Results

### Results of Evidence Synthesis

The literature review resulted in 104 papers of which 51 addressed the need for training or a curriculum with attention to 1) qualification/credentialing, 2) content of training, 3) methodology/structure of training and 4) testing/test instruments (Table II).

**Table II:** — References of literature search to define key questions for the Delphi-survey on consensus recommendations for a standardsed educational programme in robot assisted gynaecological surgery.

1.	AAGL position statement. Robotic-assisted laparoscopic surgery in benign gynecology (2013). In: Journal of minimally invasive gynecology 20 (1), S. 2–9.
2.	Advincula, Arnold P.; Wang, Karen (2009): Evolving role and current state of robotics in minimally invasive gynecologic surgery. In: Journal of minimally invasive gynecology 16 (3), S. 291–301. DOI: 10.1016/j.jmig.2009.03.003.
3.	Ahmed, Kamran; Khan, Mohammad Shamim; Vats, Amit; Nagpal, Kamal; Priest, Oliver; Patel, Vanash et al. (2009): Current status of robotic assisted pelvic surgery and future developments. In: International journal of surgery (London, England) 7 (5), S. 431–440. DOI: 10.1016/j.ijsu.2009.08.008.
4.	Asoğlu, Mehmet Reşit; Achjian, Tamar; Akbilgiç, Oğuz; Borahay, Mostafa A.; Kılıç, Gökhan S. (2016): The impact of a simulation-based training lab on outcomes of hysterectomy. In: Journal of the Turkish German Gynecological Association 17 (2), S. 60–64. DOI: 10.5152/jtgga.2016.16053.
5.	Badalato, Gina M.; Shapiro, Edan; Rothberg, Michael B.; Bergman, Ari; RoyChoudhury, Arindam; Korets, Ruslan et al. (2014): The da vinci robot system eliminates multispecialty surgical trainees’ hand dominance in open and robotic surgical settings. In: JSLS : Journal of the Society of Laparoendoscopic Surgeons 18 (3). DOI: 10.4293/JSLS.2014.00399.
6.	Bedaiwy, Mohamed A.; Abdelrahman, Mohamed; Deter, Stephanie; Farghaly, Tarek; Shalaby, Mahmoud M.; Frasure, Heidi; Mahajan, Sangeeta (2012): The impact of training residents on the outcome of robotic-assisted sacrocolpopexy. In: Minimally invasive surgery 2012, S. 289342. DOI: 10.1155/2012/289342.
7.	Brinkman, Willem; Angst, Isabel de; Schreuder, Henk; Schout, Barbara; Draaisma, Werner; Verweij, Lisanne et al. (2017): Current training on the basics of robotic surgery in the Netherlands. Time for a multidisciplinary approach? In: Surgical endoscopy 31 (1), S. 281–287. DOI: 10.1007/s00464-016-4970-2.
8.	Broholm, Malene; Rosenberg, Jacob (2015): Surgical Residents are Excluded From Robot-assisted Surgery. In: Surgical laparoscopy, endoscopy & percutaneous techniques 25 (5), S. 449–450. DOI: 10.1097/SLE.0000000000000190.
9.	Carter-Brooks, Charelle M.; Du, Angela L.; Bonidie, Michael J.; Shepherd, Jonathan P. (2017): The impact of fellowship surgical training on operative time and patient morbidity during robotics-assisted sacrocolpopexy. In: International urogynecology journal. DOI: 10.1007/s00192-017-3468-3.
10.	Choussein, Souzana; Srouji, Serene S.; Farland, Leslie V.; Wietsma, Ashley; Missmer, Stacey A.; Hollis, Michael et al. (2017): Robotic Assistance Confers Ambidexterity to Laparoscopic Surgeons. In: Journal of minimally invasive gynecology. DOI: 10.1016/j.jmig.2017.07.010.
11.	Churchill, Sara J.; Armbruster, Shannon; Schmeler, Kathleen M.; Frumovitz, Michael; Greer, Marilyn; Garcia, Jaime et al. (2015): Radical Trachelectomy for Early-Stage Cervical Cancer. A Survey of the Society of Gynecologic Oncology and Gynecologic Oncology Fellows-in-Training. In: International journal of gynecological cancer : official journal of the International Gynecological Cancer Society 25 (4), S. 681–687. DOI: 10.1097/IGC.0000000000000397.
12.	Committee opinion no. 628. Robotic surgery in gynecology (2015). In: Obstetrics and gynecology 125 (3), S. 760–767.
13.	Conrad, Lesley B.; Ramirez, Pedro T.; Burke, William; Naumann, R. Wendel; Ring, Kari L.; Munsell, Mark F.; Frumovitz, Michael (2015): Role of Minimally Invasive Surgery in Gynecologic Oncology. An Updated Survey of Members of the Society of Gynecologic Oncology. In: International journal of gynecological cancer : official journal of the International Gynecological Cancer Society 25 (6), S. 1121–1127. DOI: 10.1097/IGC.0000000000000450.
14.	Erickson, Britt K.; Gleason, Jonathan L.; Huh, Warner K.; Richter, Holly E. (2012): Survey of robotic surgery credentialing requirements for physicians completing OB/GYN residency. In: Journal of minimally invasive gynecology 19 (5), S. 589–592. DOI: 10.1016/j.jmig.2012.05.003.
15.	Gastrich, Mary Downes; Barone, Joseph; Bachmann, Gloria; Anderson, Mark; Balica, Adrian (2011): Robotic surgery. Review of the latest advances, risks, and outcomes. In: Journal of robotic surgery 5 (2), S. 79–97. DOI: 10.1007/s11701-011-0246-y.
16.	Geller, Elizabeth J.; Schuler, Kevin M.; Boggess, John F. (2011): Robotic surgical training program in gynecology. How to train residents and fellows. In: Journal of minimally invasive gynecology 18 (2), S. 224–229. DOI: 10.1016/j.jmig.2010.11.003.
17.	Göçmen, Ahmet; Sanlikan, Fatih; Uçar, Mustafa Gazi (2010a): Turkey’s experience of robotic-assisted laparoscopic hysterectomy. A series of 25 consecutive cases. In: Archives of gynecology and obstetrics 282 (2), S. 163–171. DOI: 10.1007/s00404-009-1250-6.
18.	Göçmen, Ahmet; Sanlıkan, Fatih; Uçar, Mustafa Gazi (2010b): Comparison of robotic-assisted surgery outcomes with laparotomy for endometrial cancer staging in Turkey. In: Archives of gynecology and obstetrics 282 (5), S. 539–545. DOI: 10.1007/s00404-010-1593-z.
19.	Guseila, Loredana M.; Saranathan, Archana; Jenison, Eric L.; Gil, Karen M.; Elias, John J. (2014): Training to maintain surgical skills during periods of robotic surgery inactivity. In: The international journal of medical robotics + computer assisted surgery : MRCAS 10 (2), S. 237–243. DOI: 10.1002/rcs.1562.
20.	Hoffman, Mitchel (2012a): Simulation of robotic radical hysterectomy using the porcine model. In: Journal of robotic surgery 6 (3), S. 237–239. DOI: 10.1007/s11701-011-0303-6.
21.	Hoffman, Mitchel S. (2012b): Simulation of robotic hysterectomy utilizing the porcine model. In: American journal of obstetrics and gynecology 206 (6), 523.e1-2. DOI: 10.1016/j.ajog.2012.02.001.
22.	Huser, Anna-Sophia; Müller, Dirk; Brunkhorst, Violeta; Kannisto, Päivi; Musch, Michael; Kröpfl, Darko; Groeben, Harald (2014): Simulated life-threatening emergency during robot-assisted surgery. In: Journal of endourology 28 (6), S. 717–721. DOI: 10.1089/end.2013.0762.
23.	Jarc, Anthony M.; Curet, Myriam (2015): Face, content, and construct validity of four, inanimate training exercises using the da Vinci ® Si surgical system configured with Single-Site TM instrumentation. In: Surgical endoscopy 29 (8), S. 2298–2304. DOI: 10.1007/s00464-014-3947-2.
24.	JS, Ng; al, et: Gynaecologic robot-assisted cancer and endoscopic surgery (GRACES) in a tertiary referral centre. - PubMed - NCBI. Online verfügbar unter https://www.ncbi.nlm.nih.gov/pubmed/21678011, zuletzt geprüft am 06.12.2017.
25.	Juza, Ryan M.; Haluck, Randy S.; Won, Eugene J.; Enomoto, Laura M.; Pauli, Eric M.; Rogers, Ann M. et al. (2014): Training current and future robotic surgeons simultaneously. Initial experiences with safety and efficiency. In: Journal of robotic surgery 8 (3), S. 227–231. DOI: 10.1007/s11701-014-0455-2.
26.	Lee, Yu L.; Kilic, Gokhan S.; Phelps, John Y. (2011): Medicolegal review of liability risks for gynecologists stemming from lack of training in robot-assisted surgery. In: Journal of minimally invasive gynecology 18 (4), S. 512–515. DOI: 10.1016/j.jmig.2011.04.002.
27.	Lenihan, John P.; Kovanda, Carol; Seshadri-Kreaden, Usha (2008): What is the learning curve for robotic assisted gynecologic surgery? In: Journal of minimally invasive gynecology 15 (5), S. 589–594. DOI: 10.1016/j.jmig.2008.06.015.
28.	Letouzey, V.; Huberlant, S.; Faillie, J. L.; Prudhomme, M.; Mares, P.; Tayrac, R. de (2014): Evaluation of a laparoscopic training program with or without robotic assistance. In: European journal of obstetrics, gynecology, and reproductive biology 181, S. 321–327. DOI: 10.1016/j.ejogrb.2014.08.003.
29.	Mandapathil, Magis; Teymoortash, Afshin; Güldner, Christian; Wiegand, Susanne; Mutters, Reinier; Werner, Jochen A. (2014): Establishing a transoral robotic surgery program in an academic hospital in Germany. In: Acta oto-laryngologica 134 (7), S. 661–665. DOI: 10.3109/00016489.2014.884724.
30.	Marengo, Francesca; Larraín, Demetrio; Babilonti, Luciana; Spinillo, Arsenio (2012): Learning experience using the double-console da Vinci surgical system in gynecology. A prospective cohort study in a University hospital. In: Archives of gynecology and obstetrics 285 (2), S. 441–445. DOI: 10.1007/s00404-011-2005-8.
31.	Menager, N-E; Coulomb, M-A; Lambaudie, E.; Michel, V.; Mouremble, O.; Tourette, C.; Houvenaeghel, G. (2011): Place du robot dans la formation chirurgicale initiale. Enquête auprès des internes. In: Gynecologie, obstetrique & fertilite 39 (11), S. 603–608. DOI: 10.1016/j.gyobfe.2011.07.025.
32.	Nezhat, Ceana; Lakhi, Nisha (2016): Learning Experiences in Robotic-Assisted Laparoscopic Surgery. In: Best practice & research. Clinical obstetrics & gynaecology 35, S. 20–29. DOI: 10.1016/j.bpobgyn.2015.11.009.
33.	Payne, Thomas N.; Pitter, Michael C. (2011): Robotic-assisted surgery for the community gynecologist. Can it be adopted? In: Clinical obstetrics and gynecology 54 (3), S. 391–411. DOI: 10.1097/GRF.0b013e31822b4998.
34.	Pickett, Stephanie D.; James, Rebecca L.; Mahajan, Sangeeta T. (2013): Teaching robotic surgery skills. Comparing the methods of generalists and subspecialists. In: The international journal of medical robotics + computer assisted surgery : MRCAS 9 (4), S. 472–476. DOI: 10.1002/rcs.1511.
35.	Ring, Kari L.; Ramirez, Pedro T.; Conrad, Lesley B.; Burke, William; Wendel Naumann, R.; Munsell, Mark F.; Frumovitz, Michael (2015): Make New Friends But Keep the Old. Minimally Invasive Surgery Training in Gynecologic Oncology Fellowship Programs. In: International journal of gynecological cancer : official journal of the International Gynecological Cancer Society 25 (6), S. 1115–1120. DOI: 10.1097/IGC.0000000000000466.
36.	Rossitto, Cristiano; Gueli Alletti, Salvatore; Fanfani, Francesco; Fagotti, Anna; Costantini, Barbara; Gallotta, Valerio et al. (2016): Learning a new robotic surgical device. Telelap Alf X in gynaecological surgery. In: The international journal of medical robotics + computer assisted surgery : MRCAS 12 (3), S. 490–495. DOI: 10.1002/rcs.1672.
37.	Sait, Khalid H. (2011): Early experience with the da Vinci surgical system robot in gynecological surgery at King Abdulaziz University Hospital. In: International journal of women’s health 3, S. 219–226. DOI: 10.2147/IJWH.S23046.
38.	Sananès, N.; Garbin, O.; Hummel, M.; Youssef, C.; Vizitiu, R.; Lemaho, D. et al. (2011): Setting up robotic surgery in gynaecology. The experience of the Strasbourg teaching hospital. In: Journal of robotic surgery 5 (2), S. 133–136. DOI: 10.1007/s11701-010-0231-x.
39.	Sandadi, Samith; Gadzinski, Jill A.; Lee, Stephen; Chi, Dennis S.; Sonoda, Yukio; Jewell, Elizabeth L. et al. (2014): Fellowship learning curve associated with completing a robotic assisted total laparoscopic hysterectomy. In: Gynecologic oncology 132 (1), S. 102–106. DOI: 10.1016/j.ygyno.2013.11.017.
40.	Schiff, Lauren; Tsafrir, Ziv; Aoun, Joelle; Taylor, Andrew; Theoharis, Evan; Eisenstein, David (2016): Quality of Communication in Robotic Surgery and Surgical Outcomes. In: JSLS : Journal of the Society of Laparoendoscopic Surgeons 20 (3). DOI: 10.4293/JSLS.2016.00026.
41.	Schreuder, H. W. R.; Verheijen, R. H. M. (2009): Robotic surgery. In: BJOG : an international journal of obstetrics and gynaecology 116 (2), S. 198–213. DOI: 10.1111/j.1471-0528.2008.02038.x.
42.	Schreuder, Henk W. R.; Persson, Jan E. U.; Wolswijk, Richard G. H.; Ihse, Ingmar; Schijven, Marlies P.; Verheijen, René H. M. (2014): Validation of a novel virtual reality simulator for robotic surgery. In: TheScientificWorldJournal 2014, S. 507076. DOI: 10.1155/2014/507076.
43.	Sinno, Abdulrahman K.; Fader, Amanda N. (2014): Robotic-assisted surgery in gynecologic oncology. In: Fertility and sterility 102 (4), S. 922–932. DOI: 10.1016/j.fertnstert.2014.08.020.
44.	Toptas, Tayfun; Uysal, Aysel; Ureyen, Isin; Erol, Onur; Simsek, Tayup (2016): Robotic Compartment-Based Radical Surgery in Early-Stage Cervical Cancer. In: Case reports in surgery 2016, S. 4616343. DOI: 10.1155/2016/4616343.
45.	Tunitsky, Elena; Murphy, Alana; Barber, Matthew D.; Simmons, Matthew; Jelovsek, J. Eric (2013): Development and validation of a ureteral anastomosis simulation model for surgical training. In: Female pelvic medicine & reconstructive surgery 19 (6), S. 346–351. DOI: 10.1097/SPV.0b013e3182a331bf.
46.	Vaccaro, Christine M.; Crisp, Catrina C.; Fellner, Angela N.; Jackson, Christopher; Kleeman, Steven D.; Pavelka, James (2013): Robotic virtual reality simulation plus standard robotic orientation versus standard robotic orientation alone. A randomized controlled trial. In: Female pelvic medicine & reconstructive surgery 19 (5), S. 266–270. DOI: 10.1097/SPV.0b013e3182a09101.
47.	van der PC, Sluis; al, et: [Centralization of robotic surgery: better results and cost savings]. - PubMed - NCBI. Online verfügbar unter https://www.ncbi.nlm.nih.gov/pubmed/23841923, zuletzt geprüft am 06.12.2017.
48.	Visco, Anthony G.; Advincula, Arnold P. (2008): Robotic gynecologic surgery. In: Obstetrics and gynecology 112 (6), S. 1369–1384. DOI: 10.1097/AOG.0b013e31818f3c17.
49.	Whitehurst, Sabrina V.; Lockrow, Ernest G.; Lendvay, Thomas S.; Propst, Anthony M.; Dunlow, Susan G.; Rosemeyer, Christopher J. et al. (2015): Comparison of two simulation systems to support robotic-assisted surgical training. A pilot study (Swine model). In: Journal of minimally invasive gynecology 22 (3), S. 483–488. DOI: 10.1016/j.jmig.2014.12.160.
50.	Winder, Joshua S.; Juza, Ryan M.; Sasaki, Jennifer; Rogers, Ann M.; Pauli, Eric M.; Haluck, Randy S. et al. (2016): Implementing a robotics curriculum at an academic general surgery training program. Our initial experience. In: Journal of robotic surgery 10 (3), S. 209–213. DOI: 10.1007/s11701-016-0569-9.
51.	Witkiewicz, Wojciech; Zawadzki, Marek; Rząca, Marek; Obuszko, Zbigniew; Czarnecki, Roman; Turek, Jakub; Marecik, Sławomir (2013): Robot-assisted right colectomy. Surgical technique and review of the literature. In: Wideochirurgia i inne techniki maloinwazyjne = Videosurgery and other miniinvasive techniques 8 (3), S. 253–257. DOI: 10.5114/wiitm.2011.33761.

***Qualification/Credentialing***

Of 51 papers selected, 25 contained credentialing recommendations. Some authors advised against definitions for a centre to be accredited (Erickson et al., 2012). Others underlined that by avoiding such clarity, centres cannot evade their responsibility for correct introduction and safe use of robotic surgery systems (Pradarelli et al., 2017). These authors argued that training standards promoted by manufacturers were insufficient. In contrast, surgeons and hospitals were obliged to develop educational strategies to keep up with new surgical advances while considering their duty of care to patients.

Institutions are responsible for governance, including repetitive re-assessments to maintain surgical privileges (Committee opinion no. 628, 2015). A recent Canadian study showed considerable variation among institutions and standardisation proved difficult, but necessary (Siddique et al., 2016). The recognition of ‘centres of excellence’ might assist although criteria for such centres are not defined. In general, it is assumed that high volume units qualify as training centres (Gastrich et al., 2011; Gobern et al., 2013).

***Content of training, including courses***

**Figure 2:**
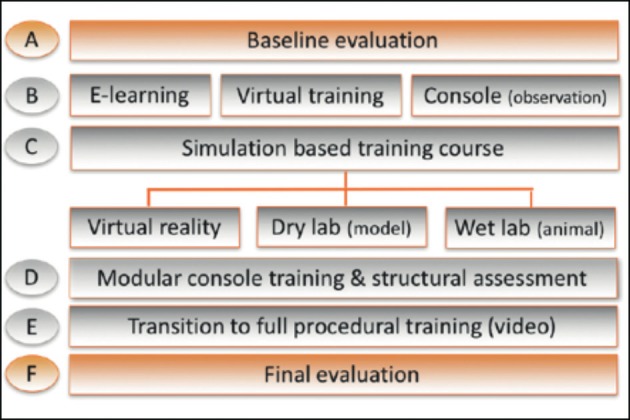
SERGS curriculum (modified for gynaecology after Volpe et al., 2015).

Forty-five of the 51 papers addressed this issue. Structured and standardised training with pre-set learning goals is paramount to accomplish training in a timely and thorough fashion (Geller et al., 2011). Modules (see Qualification/Credentialing) of training lead from e-learning, to virtual training, to model training, and finally to procedural training (Figure 2, modified for gynaecology after Volpe et al. (2015)).

E-learning tools are considered as a basis for basic and advanced training (Maertens et al., 2016). In later stages of practice, e-learning may provide a resource for permanent training by sharing information provided by professionals themselves (www.websurg.com; https://eacademy.esgo.org/). Virtual training may teach technical skills in a simulated and safe environment and provide tools for objective assessment. Validated systems are commercially available (Abboudi et al., 2013; Moglia et al., 2016).

Construct validation (whether the exercise is discriminatory) and face validation (whether the exercise resembles real-life) need to have been assessed (Schreuder et al., 2014).

Model training may teach technical skills in a more realistic environment by working in a box trainer, an animal model, or a cadaver (Sridhar et al., 2017). An important and final part is procedural training in virtual and in-vivo procedures. Clinical procedures should be performed under the guidance of expert tutors (Sluis van der et al., 2013; Sandadi et al., 2014; Sridhar et al., 2017). In the ERUS experience, a modular sequential introduction to complex procedures was the safest and most effective way to learn complex surgery. Rather than starting and finishing a whole procedure at once, modular training takes the trainee stepwise through well-defined structured stages. This approach ensures maximum attention for each step, avoiding concentration loss during long procedures with multiple parts (Crane et al., 2013; Letouzey et al., 2014; Lovegrove et al., 2016; Carter-Brooks et al., 2018).

***Methodology/Structure of training***

Of the 51 papers, 33 included guidance for structured tuition, either in modular training (n=11), stepwise learning (n=7), or both (n=15).

Training in complex procedures using sophisticated technology requires systematic, structured and (therefore) modular training (Schreuder and Verheijen, 2009; Ng et al., 2011; Letouzey et al., 2014). This has been developed and validated by ERUS for prostatectomy (Volpe et al., 2015). The literature search showed a plea for curricula to be built up from e-learning, through virtual and box training to artificial and animal model teaching (see also Content of training, including courses) (Volpe et al., 2015).

***Testing/Test Instruments***

Not all papers that discussed the content and structure of training defined relevant and measurable end points. Only 29 of the 51 papers gave recommendations for assessment of training.

*Competency based assessment*

Competence based training with structural assessment has been introduced in the curriculum for general gynaecology successfully (Boerebach et al., 2016) . The Royal College of Physicians and Surgeons of Canada described seven competencies of a physician which included ‘professional’, ‘communicator’, ‘collaborator’, ‘leader’, ‘health advocate’, ‘scholar’, and ‘medical expert’ as central roles (Frank et al., 2015). Evaluation of these roles is now integrated in the assessment of general obstetrics and gynaecology training in the United Kingdom and Netherlands (Garofalo and Aggarwal, 2017). In a technical field such as robotics, these competencies are essential for a future expert and team to evolve (Sananès et al.,2011; Payne and Pitter, 2011; Witkiewicz et al., 2013; Schreuder et al., 2014).

*Structured assessment*

A regular, non-judgemental and objective evaluation of progress is regarded essential for effective learning and patient safety. Pre- and post-testing at various modules help to develop skills (Thomaier et al., 2017).

Systematic assessment after each module or parts thereof should monitor progression. Structured assessment enables the tutor to systematically review skills and competencies. Objective and quantitative scoring can be performed using the Global Evaluative Assessment of Robotic Skills (GEARS) (Goh et al., 2012) and Objective Structured Assessment of Technical Skills (OSATS) (Faulkner et al., 1996). GEARS is the only instrument designed and validated for robot-assisted surgery (Sánchez et al., 1996; Goh et al., 2012).

To integrate non-technical competencies Non-technical Skills for Surgeons (NOTSS) has been developed (Flin et al., 2006). At the end of training, assessment of an unedited video of a procedure performed by the trainee should be part of a final evaluation (Payne and Pitter, 2011; Hoffman et al., 2012a,b; Vaccaro et al., 2013). This allows appraisal by an independent assessor using tools like GEARS (Tunitsky et al., 2013). Video assessment is now offered commercially to monitor the performance of individual robotic surgeons (White et al., 2015; Polin et al., 2016).

*Volumetric criteria*

The portfolio with subsequent assessments avoids defining a volume criterion for certification. Various studies have resulted in volume criteria that range from 10 to 100 procedures necessary to reach proficiency (Pitter et al., 2008; Brinkman et al., 2012; Letouzey et al., 2014; Sinno and Fader, 2014; Conrad et al., 2015; Ring et al., 2015; Nezhat and Lakhi, 2016; Brinkman et al., 2017). However, certification should not be based on numbers only but predominantly on assessment of competence (Brinkman et al., 2012, 2017).

### Results of the Delphi process

Consensus was reached in multiple areas of robotic education, qualification, course and content of training, structure of curriculum, and assessment tools (Table 1). Among all panel members there was agreement that a standardised training curriculum for gynaecology would be advantageous for robotic assisted gynaecological surgery (Q1).

### Qualification

*Requirements for the trainer/proctor*

Consensus was reached that trainers should be accredited. There were no suggestions on the content and instruments for trainer-certification nor on its implementation (Q2).

*Requirements for the Educational Training Centre*

Consensus was reached that training centres should be accredited by a recognised society (Q7). Agreement was reached that accreditation of centres should be based on case volume (Q8) and expertise of the trainer (Q9). Although hard data are lacking, the panel agreed on a minimal requirement of over 100 cases/year per center (Q9) as a prerequisite for accreditation.

*Requirements for qualification as an independent surgeon*

Consensus was reached on a minimum of ten mentored cases before a trainee should work independently (Q4). Furthermore, experienced surgeons should continue to be tested on the advanced curriculum (Q3), although this issue was not part of the reviewed statements. To qualify for certification, a video of the index procedure in addition to a completed logbook (Q39) should be submitted to the society for review.

It was felt that experienced surgeons did not need to be assessed in advanced procedural training if they were familiar with their platform (Q2). However, there was a vote for trainers to have basic training if they changed platforms (Q2).

Whilst there was consensus that surgeons continue to report their outcomes after certification, there was no consensus on a reporting template to be used (Q6). In general, it was recommended to include patient specific details, comorbidities, BMI, operative details, length of stay, preoperative staging, operation time, pathological staging, readmissions, and complications using the Clavien-Dindo classification (Clavien et al., 2009) (Q10).

### Course/Content of Training Curriculum

*Modular training*

In line with the literature, consensus was reached that educational curricula for robot-assisted gynaecological surgery should be in stages (Q11), each with theoretical and practical exams. Trainees should pass each module before commencing the next (Q17).

*Basic training*

Basic training should include baseline evaluation, e-learning, simulation based training, procedure observation, and team simulation (Q12).

- A baseline evaluation should help group novices by their theoretical knowledge and pre-existing skills. For this purpose, a written test and VR-simulation were recommended (Q13).
- E-learning should include information on patient selection, port placement, docking, trouble shooting, link to FRS, theatre team efficiencies, non-technical skills and standardised emergency management (Q14). It was recommended that required operating room observations should be volume based (Q15).
- Basic simulation training should include VR-simulation and dry- and wet-lab teaching (Q16).
- Team training should include emergency scenarios, team decision making, bedside assistance, docking and patient turnaround (Q21). Among the CanMed roles – which SERGS subscribes to - leadership is regarded as an important non-technical-skill (Frank et al., 2015). No consensus was reached on recommendations for the content of non-technical skills training (Q20).

*Advanced training*

Analogous to basic learning, consensus was reached to perform advanced training in a modular/stepwise approach. E-learning on index procedures was recommended supplemented by video demonstrations, access to video libraries, simulation, modular console teaching, transition to full training, and a final evaluation (Q18).

It was agreed that advanced e-learning in a modular, stepwise approach should also take into account aspects such as patient selection and preparation, port placement, non-SERGS consensus on robotic training technical skills training, trouble shooting, emergency scenario management and knowledge of additional equipment in theatre (Q19).

### Structure of Curriculum

Standardisation of educational programmes seems necessary to compare outcomes. In this context, specific aspects of the structure of a robotic curriculum were reviewed.

*Target Group*

It was felt that consideration of prior knowledge and experience was important (Q22). Consensus was reached that there should be a common approach for basic robotic skills training with similar pathways across subspecialties (Q23).

*Target Skills*

Index procedures mastered over a given time should be suitable as proof of general theoretical knowledge and practical skills of a novice (Q25). A simple hysterectomy (Q26) was deemed an appropriate index procedure for benign gynaecology and a pelvic lymphadenectomy (Q27) for gynaecological oncology. A stepwise approach (modular training) of the index procedure was regarded advantageous (Q24). It seemed necessary to have a proctor present when the trainee transitioned to full procedures in his/her institution (Q28).

### Test Instruments

A validated scoring system is beneficial for the trainee to provide consistent feedback (Q33). Such instruments should have been tested for face- and construct-validity. Questions may be used as instruments for testing knowledge at each educational level (Q29).

For testing theoretical knowledge on different educational levels the panel agreed that

- online tests are suitable instruments for evaluating progress for advanced learning modules (Q30).
- efficiency of non-technical-skills training should be assessed with a scoring system (Q31). The use of the NOTSS-System is recommended (Q32).

For assessment of procedural progress it was advised to

- evaluate the full operation with a submitted video (Q34) by two certified “expert” independent examiners (Q38). No consensus was reached on case numbers to be sent for analysis (Q35). The survey did not address criteria for certifying an examiner.
- use of validated scoring systems to analyse videos (Q36). Simultaneously, the panel could only make a general recommendation for the use of subjective and objective scoring systems (Q37).

## Discussion

The Delphi method structures group communications to process complex problems (Thangaratinam, 2005; Collins et al., 2016). It is used to gain and aggregate expert opinions on issues “where hard data is unavailable” (Mahajan et al., 1976). Seven members are considered as “a suitable minimum panel size” for a Delphi-process, but sizes vary between 4 to 3000 (Mahajan et al., 1976; Thangaratinam, 2005). In the end, the panel size will be subject to the availability of dedicated experts. In the context of minimal invasive gynaecological surgery the Delphi-method has been used to define assessment of laparoscopic gynaecological procedures such as hysterectomy (Tremblay et al., 2014).

We used the results of a literature search as a backbone for formulating topics of discussion, called herein issues. Through the Delphi process, major issues in training of robotic surgeons were identified and the minimal requirements agreed. In summary, identification of training centres is volume based, next to the availability of educational tools such as e-learning, virtual learning, model training and supervised procedural teaching. The training programme should be modular, with regular assessments to monitor progress. Unlike classical surgical training, procedural learning should be stepwise allowing a focus on each step. A portfolio should help adherence to systematic training and assessment and provide the basis for certification.

The introduction of systematic and structured learning has changed surgical training. The ‘see one, do one, teach one’ principle has been abandoned and assessment of surgical performance is no longer a short observation by a single tutor resulting in a brief and undocumented verdict. During training, not only technical skill is important but also other competencies are recognised as valuable for medical education and these need assessment (Frank et al., 2015). Competence based assessment is now accepted, and urologists have embraced this for robotic training (Ahmed et al., 2010). Although the Delphi consensus did not result in abandoning volume based criteria for certification, competence based assessment places the emphasis on proficiency. The evolution of competence is assessed in the portfolio.

Risks to patients during an apprenticeship can be minimised by stepwise training with hands-on learning in a dry and wet laboratory before embarking on a real-life procedure. E-learning modules have been developed to prepare for hands-on training (Maertens et al., 2016). Virtual training modules have been developed for technical and procedural training (Julian et al., 2018). Box training for technical instruction and development of hand-eye co-ordination has been validated (Stefanidis et al., 2011). Finally, performance during real-life surgery can be evaluated objectively using assessment tools such as OSATS (Faulkner et al., 1996).

There are several drawbacks of the process leading to the development of a SERGS’ training programme. Firstly, the size of the expert panel is small because there are relatively few gynaecological surgeons regularly using a robot and being involved in training in this new technique, the number of panel member numbers is low. At the same time this reflects the urgency of such a curriculum in order to promote expertise in the robotic field. As issues addressed were also reviewed in the literature, this was not felt to cause major bias. Secondly, the literature on training in gynaecological robot-assisted surgery is limited. The general principles of medical education also apply to specific training in robot-assisted surgery. Therefore, these principles were included also to obtain a representative view of surgical training.

In preparation, SERGS drafted a pilot curriculum in the form of a fellowship-programme with four robotic novices trained in four high-volume centres of excellence (Rusch et al., 2018). The curriculum was standardised with a modular and stepwise educational programme and used validated tests as proof of efficacy. This limited experience proved a need for more in depth evaluations of various educational issues, as well as the need for close monitoring of curriculum adherence. In particular, it revealed trainers were generally unacquainted with educational tools and should be trained themselves.

This Delphi process provides minimal requirements for a suitable programme. It has been the basis for the SERGS endorsed curriculum with clear outlines of training needs including assessment tools. For the index procedures, it details steps that need to be taught (Supplementary Material, Appendix 1; link: https://www.sergs.org/wp-content/uploads/2015/08/SERGS-Curriculum-Final.pdf). This curriculum needs validation but could be used without because the Delphi process defined minor variations only in the recently validated ERUS curriculum (Volpe et al., 2015).

Finally, it should be acknowledged that this process of curriculum development has not touched on the issue of maintenance of profiency and gouvernance, neither on the issue of training of the trainers (Collins et al., 2019). This needs to be developed to secure excellent and safe care of our patients.

## Conclusion

In conclusion, immediate implementation of a structured curriculum is recommended. Guidance for training is needed as stricter regulation and monitoring of surgeons is demanded. There is increasing awareness that the safe introduction of new technology is the responsibility of individual institutions and care providers (Lee et al., 2011; Pradarelli et al., 2017). Guidance will assist implementing standardised and adequate educational programs.

## Appendix 1

SERGS CURRICULUM

for robot assisted gynaecological surgery.

Final Curriculum (approved by SERGS council 30.09.17).
